# Characteristics of successful termination of atrial fibrillation by atrial antitachycardia pacing in patients with cardiac implantable electronic devices

**DOI:** 10.1007/s00380-024-02409-2

**Published:** 2024-04-28

**Authors:** Yoshiyasu Aizawa, Satoru Komura, Emiko Kawakami, Shonosuke Watanabe, Kazuki Tanaka, Hiromu Kadowaki, Atsushi Takagi

**Affiliations:** 1 Department of Cardiovascular Medicine, Nippon Medical School Hospital, 1-1-5, Sendagi, Bunkyo-ku, Tokyo 113-8603 Japan; 2 Komura Cardiovascular Clinic, Saitama, Japan; 3 Department of Cardiology, Saiseikai Kawaguchi General Hospital, Saitama, Japan

**Keywords:** Pacemaker, Implantable cardioverter defibrillator, Atrial fibrillation, Atrial antitachycardia pacing

## Abstract

Asymptomatic paroxysmal atrial fibrillation (AF) is often found in patients implanted with cardiac implantable electronic devices (CIEDs). Second-generation atrial antitachycardia pacing (A-ATP) is effective in managing AF in patients implanted with CIEDs. The purpose of this study was to evaluate the efficacy and safety of A-ATP in patients implanted with CIEDs. This was a single-center retrospective study involving 91 patients (male 46 patients, mean age 74 ± 9 years) implanted with Reactive A-ATP equipped devices (84 patients with pacemakers, 6 with ICDs, and 1 with a CRT-D). The AF burden, rate of AF termination, and details of the activation of the A-ATP were analyzed in each patient. During a mean follow-up period of 21 ± 13 months, A-ATP was activated in 45 of 91 patients (49.5%). No patients had adverse events. Although the efficacy of the A-ATP varied among the patients, the median rate of AF termination was 44%. In comparison to the A-ATP start time, “0 min” had a higher AF termination rate by the A-ATP (39.4% vs. 24.4%, *P* = 0.011). The rate of termination by the A-ATP was high for AF with a long cycle length and a relatively regular rhythm. A-ATP successfully terminated AF episodes in some patients implanted with CIEDs. The optimal settings of the A-ATP will be determined in future studies.

## Introduction

Atrial fibrillation (AF) is the most common sustained arrhythmia and the prevalence of AF increases with advanced age [1]. Thromboembolisms including strokes are the most important complication of AF [2]. Detection of AF by cardiac implantable electronic devices (CIEDs), which constantly monitor the occurrence of AF, has a high sensitivity [3, 4]. Asymptomatic AF detected by CIEDs is associated with an increased risk of thromboembolic complications including strokes [5].

**Fig. 1:**
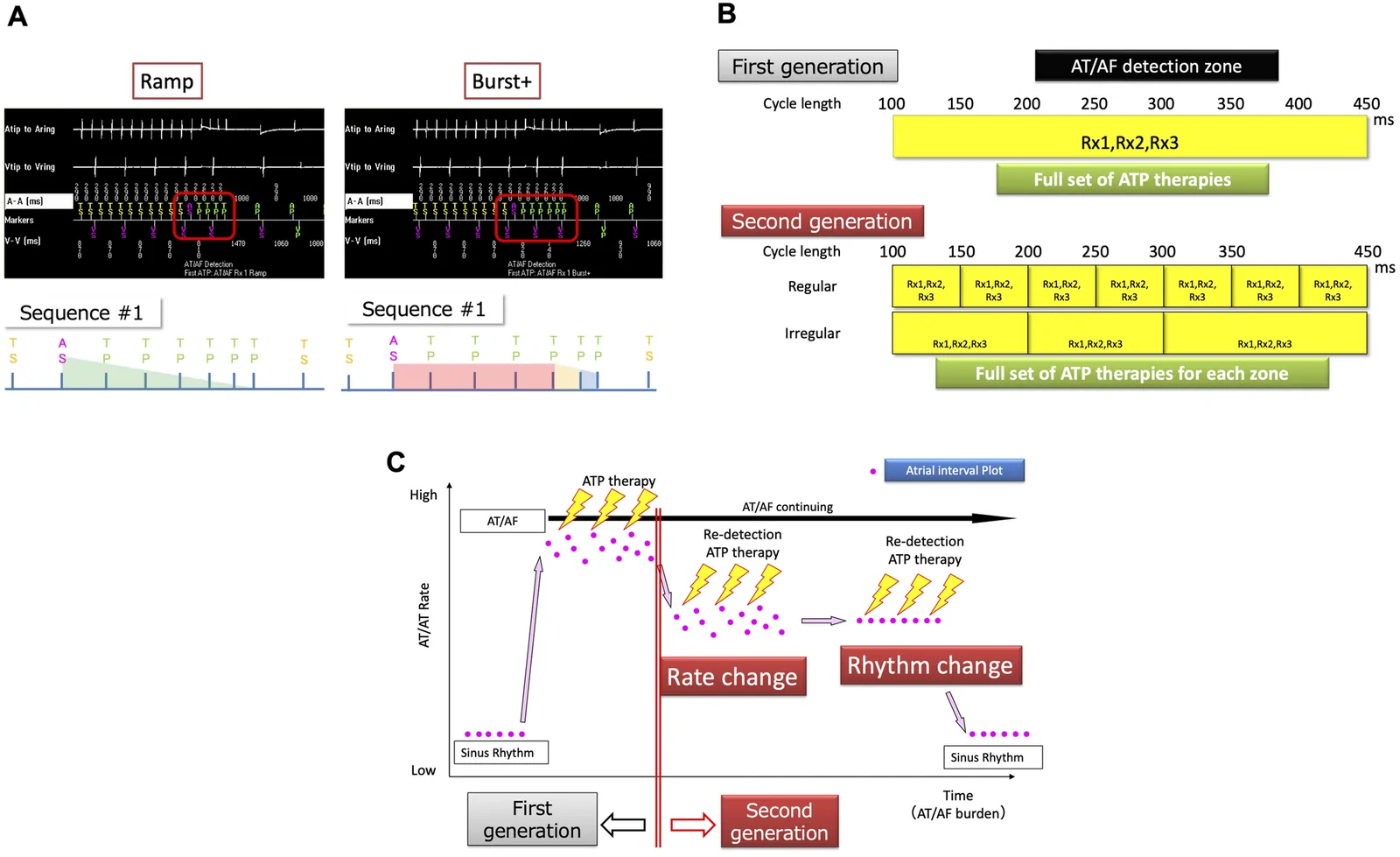
The A-ATP and reactive ATP algorithm. **A** There are two types of A-ATP: ramp pacing (left) and burst + pacing (right). In the nominal device settings, Tx1 is ramp and Tx2 is burst + . **B** Difference between the 1st and 2nd generation A-ATP. In the first-generation A-ATP, the session ended after the completion of the number of A-ATP therapies programmed. The current second-generation reactive ATP continues to monitor AF after AF termination has failed. When a rate or rhythm change is triggered for some reason, A-ATP is resumed. A change in the AF rate or rhythm indicates an unstable state of AF. Therefore, administering A-ATP at this time can terminate AF. **C** Reactive ATP algorithm. Improving the rate of termination of atrial fibrillation by administering A-ATP at the moment the rate and rhythm of the atrial fibrillation change

Reactive ATP (Medtronic Inc., Minneapolis, MN, USA) is a function of CIEDs that allows for multiple deliveries of programmed atrial antitachycardia pacing (A-ATP) therapies for atrial tachyarrhythmias. Reactive ATP attempts to terminate atrial tachyarrhythmias in response to a change in the atrial rhythm’s cycle length or regularity (Fig. 1). The MINERVA randomized multicenter international trial reported that reactive ATP was associated with a reduction in the progression of atrial tachyarrhythmias to permanent AF [6]. However, there are little real-world data regarding the efficacy of reactive ATP in the clinical situation. Therefore, we conducted a retrospective observational study to investigate the characteristics of the efficacy of reactive ATP in patients implanted with CIEDs.

## Materials and methods

This was a retrospective observational study performed at a single hospital. We investigated 91 consecutive patients after the implantation of CIEDs equipped with reactive ATP (Medtronic: Advisa DR, Advisa DR MRI, Evera XT DR, Viva Quad XT CRT-D) from January 2013 to December 2016. The mean age of the patients was 74 ± 9 years and 46 patients were male. The indications for those devices are shown in Table 1. Among 91 patients, 34 patients had a history of paroxysmal AF and 57 patients did not have a history of AF before device implantation. Written informed consent was obtained from all patients before any invasive procedures regarding inclusion for observational clinical studies. This study was approved by the ethics committee of Saiseikai Kawaguchi General Hospital and have, therefore, been performed in accordance with the ethical standards laid down in the 1964 Declaration of Helsinki and its later amendments.

**Table 1:** Patient background

Devices	Etiology	Patients
Pacemakers		
	SSS	48 patients
	AV block	36 patients
ICDs		
	OMI + VT	4 patients
	DCM + NSVT	1 patient
	HCM + VT	1 patient
CRT-D	Myocarditis	1 patient
Total		91 patients

*SSS* sick sinus syndrome; *AV* atrio-ventricular; *OMI* old myocardial infarction; *DCM* dilated cardiomyopathy; *VT* ventricular tachycardia; *NSVT* non-sustained ventricular tachycardia; *HCM* hypertrophic cardiomyopathy; *CRT-D* cardiac resynchronization therapy defibrillators

**Fig. 2:**
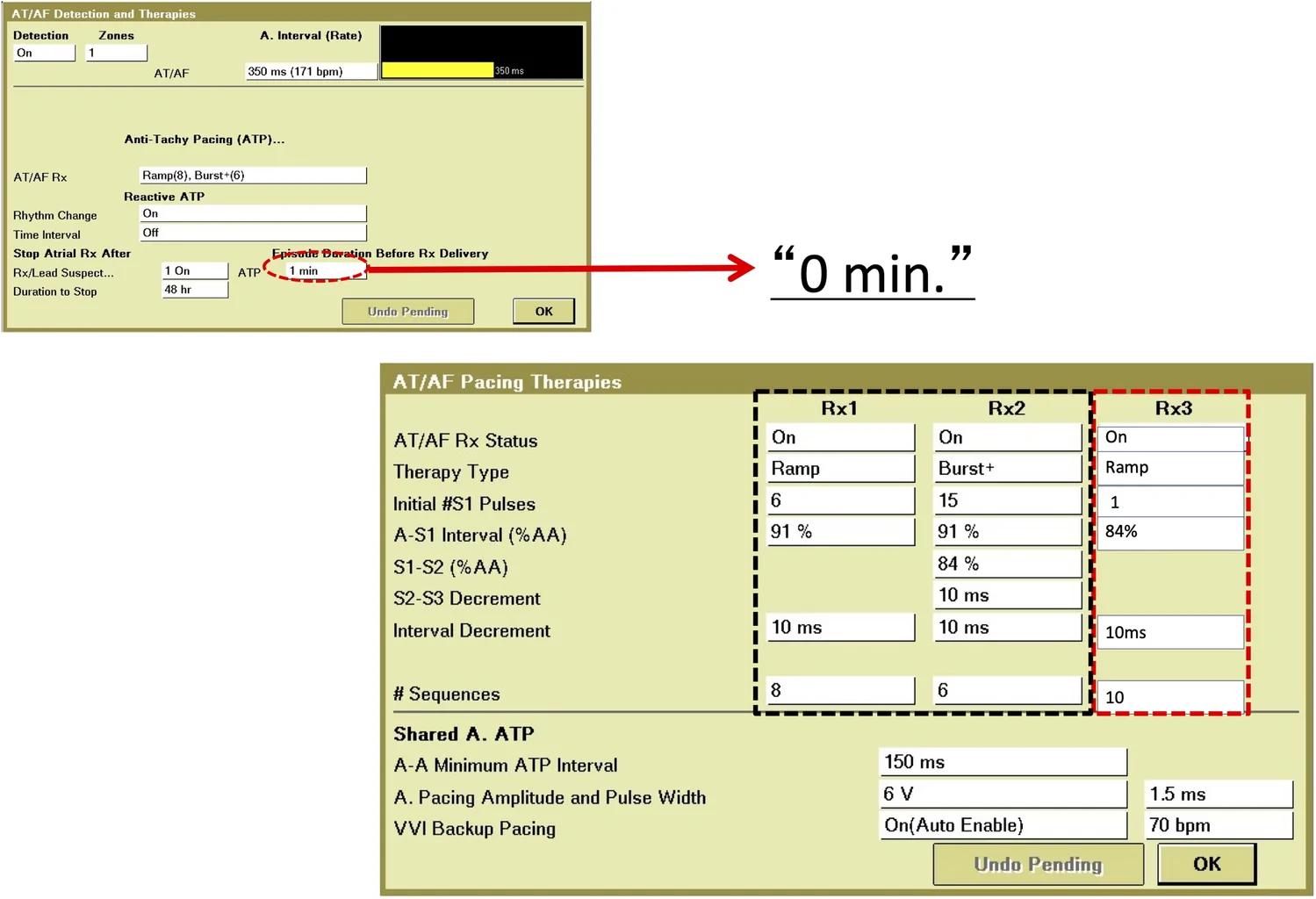
Reactive ATP settings in this study. The nominal settings were used for AF detection. In the nominal settings, A-ATP is initiated 1 min after AF detection, but in this study, A-ATP was programmed to start immediately after AF detection (= 0 min)

Reactive ATP was programmed in those patients. The ATP settings were programmed as follows: the atrial tachyarrhythmia (ATA) detection interval was set at ≤ 350 ms and a delay of 0 min or 10 min between the onset of the ATA and ATP delivery was programmed. The ATA zone was subdivided into a series of narrower sub-zones of 50 ms: a length from a 100 to 350 ms subzone for regular rhythms and into three subzones for irregular rhythms (100–200 ms, 200–300 ms, and 300–350 ms). Each subzone was supplied with a separate set of atrial ATP therapies that were 10 ramp sequences, 10 burst + sequences, and 10 ramp sequences (Table 2). If the rhythm shifted into a different subzone due to a change in the cycle length or regularity, the device delivered therapies from those available in the new subzone (Fig. 2). An ATP therapy was considered successful if sinus rhythm was obtained within 20 s after the last ATP attempt. The ATP program was activated on the day of the first clinic follow-up visit after the device implantation. The trial leads were placed in the right atrial appendage in all patients.

**Table 2:** Reactive ATP programming

Therapy sequence	Rx1	Rx2	Rx3
Therapy	Ramp	Burst +	Ramp
Initial pulse number	13	11	13
A-S1	91%	84%	81%
S1S2		81%	
S2S3		20 ms	
Dec interval	10 ms	10 ms	10 ms
Sequence number	10	10	10

Clinical data were collected from all patients including the age, gender, underlying heart disease, and implanted devices. The number of ATA episodes, AF burden, and AF termination rate during the follow-up period were also collected from all patients. In the ATP group, the number of ATP deliveries and the success rate of the ATP were evaluated. The patients were followed up every 3–6 months for a clinical review and device interrogation.

### Statistical analysis

Continuous variables are presented as the mean ± SD and categorical variables as numbers and proportions. Continuous variables were compared using a Mann–Whitney *U* test. Categorical variables were compared between groups using the chi-square test. Statistical analyses were performed on IBM SPSS Statics software (Version 29). A *P* value of < 0.05 was considered as statistically significant.

## Results

**Fig. 3:**
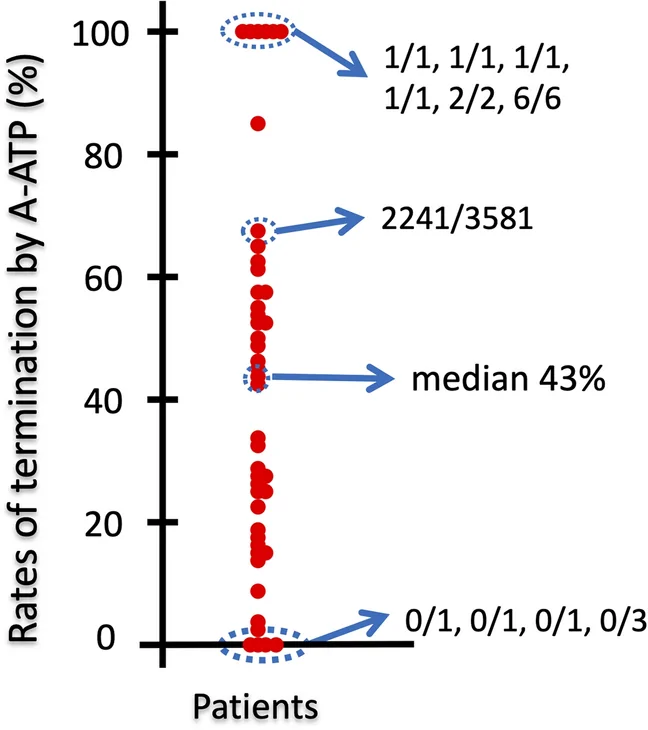
Termination rate by A-ATP in each patient. Numbers indicate successful termination/attempt of A-ATP. The median rate of termination by A-ATP in all patients was 43%. Maximum 3581A-ATPs were operated on 1 patient and termination rate was 61.8%. Note the extremely high and low termination rates were due to the fewer attempts of A-ATP

**Fig. 4:**
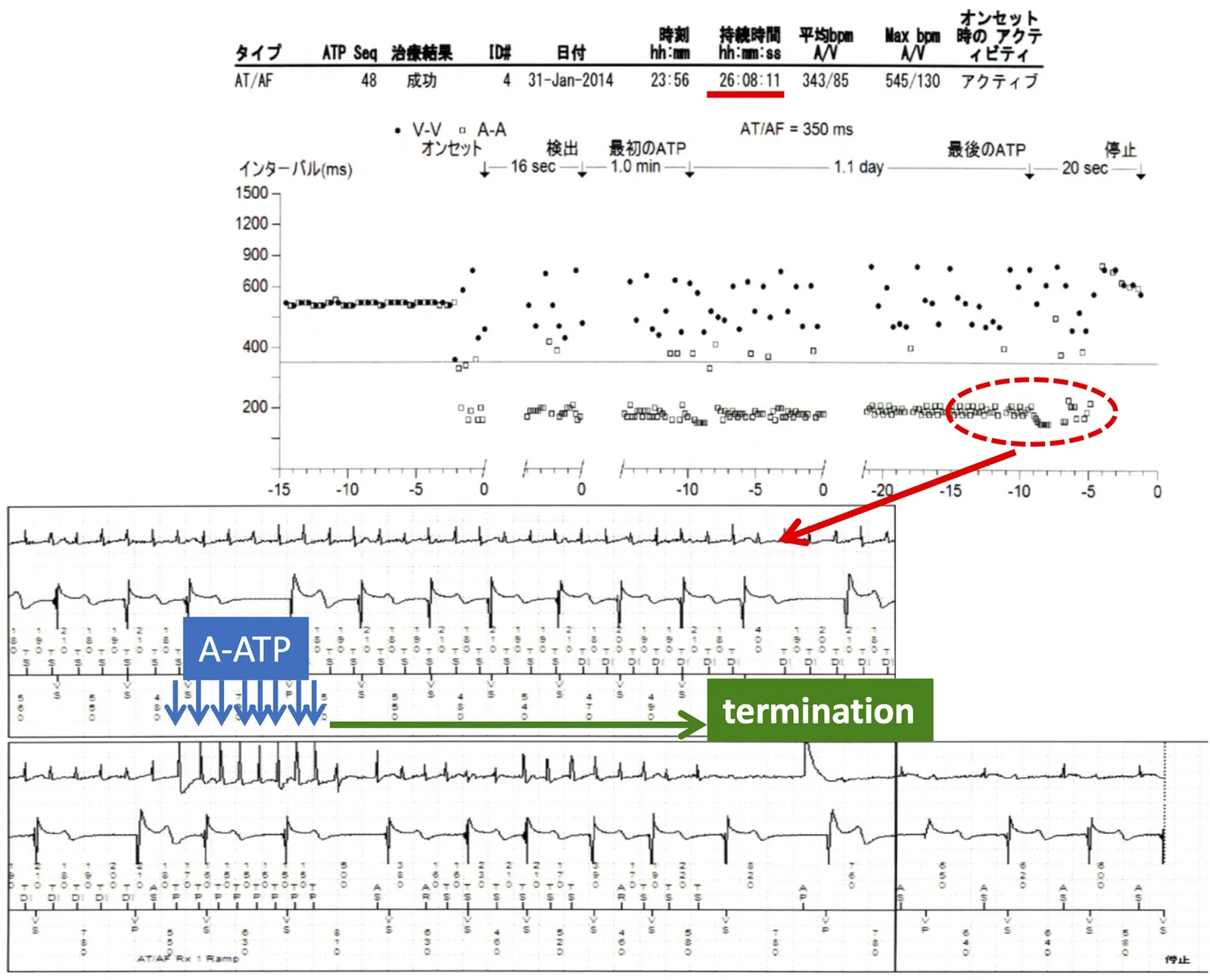
Representative case of AF termination by reactive ATP. A 79-year-old male had paroxysmal AF lasting about 26 h, and a total of 48 A-ATP attempts were delivered and the AF was terminated. Note the AF was terminated 3 s after the A-ATP delivery

**Fig. 5:**
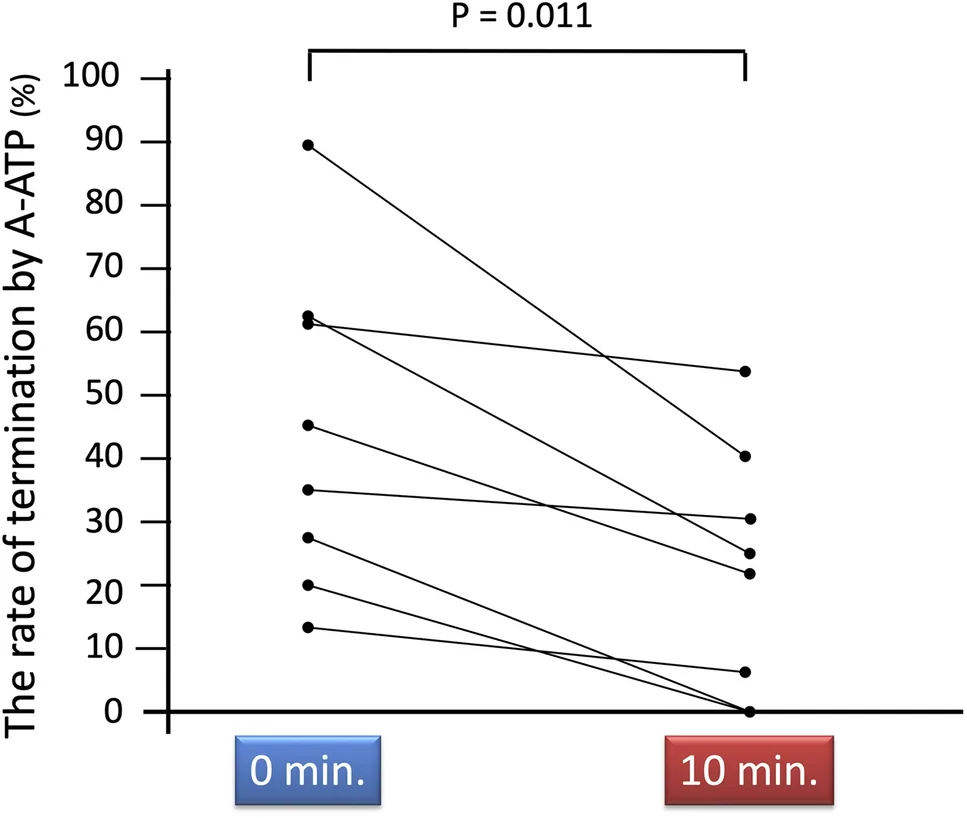
The superiority of early intervention for the AF termination rate. The rate of AF termination was significantly higher for “0 min” as compared to that for “10 min” (39.4% vs. 24.4%, *P* = 0.011)

An A-ATP activation was observed in 45 of 91 cases (49.5%) during a mean follow-up period of 21 ± 13 months. Ten cases (11.0%) were newly diagnosed with AF after device implantations. Figure 3 shows the termination rate of AF by A-ATP and it can be seen that there were very large individual differences in the termination rate. The termination rate of 0% or 100% was thought to be due to the small number of episodes occurring. The median termination rate was 43%. There were 22 out of 45 cases (48.9%) with a termination rate of 44% or higher. The highest number of episodes reached 3581 episodes, and the termination rate was 61.8%. Furthermore, no cases of progression to chronic AF or adverse events due to A-ATP were observed. The longest episode of AF, which was terminated by A-ATP, was approximately 26 h. In most episodes of AF termination, the AF termination was obtained not during A-ATP but several seconds after the A-ATP attempt (Fig. 4). Since, we applied the nominal A-ATP start time of “10 min” (default) at the beginning and changed it to “0 min” from a certain time for all patients. We could compare the efficacy of A-ATP in 8 patients which changed A-ATP start time from “10 min” to “0 min”. In a comparison of the A-ATP start time, a “0-min” pattern had a higher AF termination rate by A-ATP than that of the start time, “10 min” (39.4% vs. 24.4%, *P* = 0.011, Fig. 5). AF burden was shortened in 6 out of 8 patients by changing A-ATP start time from “10 min” to “0 min”. AF burden tended to be shorter in “0 min.” than in “10 min” but not statistically significant (0.54 h/day vs. 0.74 h/day, *P* = 0.34, not shown).

**Fig. 6:**
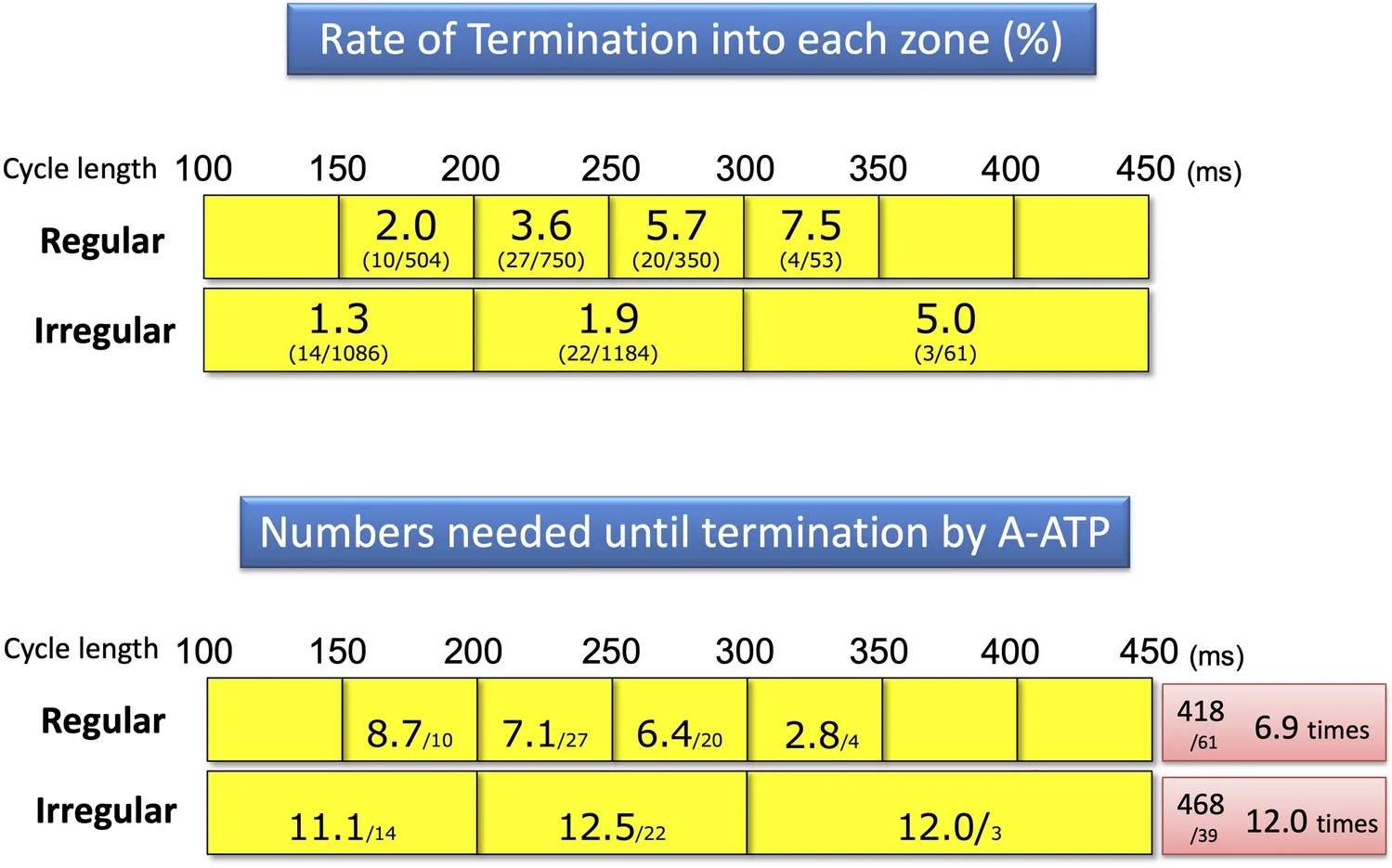
Efficacy of the reactive ATP. Upper row: The termination rate of AF was analyzed for each zone. The termination rate was higher for regular AF than the irregular zones. The termination rate of AF was higher at a slower atrial rate than that of a fast atrial rate. Lower row: The numbers needed until achieving termination of AF were analyzed for each zone. The numbers needed for termination by ATP were fewer for the zones with regular and slower atrial rates

Figure 6 shows the characteristics of the AF, which was terminated by A-ATP. The results showed that the termination rate was higher in the regular zone than in the irregular zone and that the termination rate was higher in the slow atrial rate zone than fast atrial rate zone.

## Discussion

It has been more than 60 years since the first implantable pacemaker was developed, and to date, basic improvements have been made to the pacemaker device, such as making the device smaller, improving the durability, including of the electrode leads, and extending the battery life. In recent years, MRI-compatible pacemakers have become standardized, and leadless pacemakers are becoming more widespread. In addition, there have been remarkable developments in the functionality of pacemakers [7].

The ASSERT study reported the frequency of AF and the risk of thromboembolisms in 2580 patients aged 65 years or older without a history of AF who had device implantations including pacemakers. According to the report, AF that lasted for more than 6 min was observed in 10.1% of patients within 3 months after the device implantation and in 34.7% over 2.5 years [8]. Furthermore, patients with AF had a relative risk of thromboembolisms 2.49 times (1.28–4.85, *P* = 0.007) that of patients without AF. Additionally, in the SOS-AF study, based on the episode records of more than 10,000 pacemaker patients, the risk of a thromboembolism was doubled in patients with a cumulative AF time of 1 h or more per day [9]. Those reports suggest that asymptomatic AF may be extremely common in patients with pacemakers, and early intervention would be important in those patients to prevent thromboembolisms.

Various pacemaker functions have been developed to suppress AF by pacemaker algorithms, but none of the functions have been able to demonstrate their effectiveness. AF is thought to be caused by a complex interplay of a wide variety of factors, including remodeling and inflammation of the atrial muscle due to aging or a high blood pressure and the influence of sympathetic and parasympathetic nerves. Therefore, it is extremely difficult to suppress AF occurrences using only the pacing function. On the other hand, A-ATP is a function that does not aim to suppress the occurrence of AF, but rather to stop the AF that has already occurred. This feature is installed in a variety of cardiac devices, including pacemakers manufactured by Medtronic.

So far, clinical studies that tested the effectiveness of the first-generation A-ATP over 10 years ago failed to demonstrate its effectiveness [10]. However, its effectiveness was demonstrated with the advent of the second-generation A-ATP, which added the reactive ATP function. The crucial difference between the first and second generations is that the first-generation A-ATP was an algorithm that performed A-ATP only a set number of times when AF was detected. The second generation of A-ATP focuses on the fact that the atrial rate interval changes (= rate change) and the regularity of the atrial rate changes (= rhythm change) while AF continues. The algorithm has been developed to create 10 zones based on the atrial rate and its regularity, and repeatedly perform A-ATP at the moment when AF shifts to each zone. This function is collectively called reactive ATP. In 2014, the MINERVA trial was reported and verified the effectiveness of the second-generation A-ATP [6]. The results showed that compared to the control group, the composite endpoint (death, cardiovascular hospitalization, and progression to chronic AF) had reduced in the A-ATP group by 26% and the progression to chronic AF by 61%. It also reduced the risk of AF lasting more than one day by 34%, and the risk of AF lasting more than seven days by 48%. Real-world data from a large device database composed of 43,440 patients showed that the reactive ATP was significantly associated with a reduced risk of AT/AF [11]. Ueda et al. [12] reported that reactive ATP successfully and safely reduced the AF burden, hospitalizations due to heart failure, and ventricular arrhythmias in patients implanted with CRTs. Sumiyoshi et al. examined whether the AF burden varies according to the pacing site and compared high atrial septal pacing and right atrial appendage pacing. The incidence of an AF lasting longer than 1 or 7 days and the median time of AF/AT per day was significantly lower in high atrial septal pacing than atrial appendage pacing group [13]. In our study, the termination rate by the A-ATP was higher for AF with a long cycle length and a relatively regular rhythm. It could be explained that pacing stimulation from the atrium RA easily reaches into the reentrant circuit with a regular or slower rate rather than that with an irregular or faster rate [14]. However, the dominant frequency of AF is usually higher in the LA than in the RA [14]. Analysis of the LA is insufficient in our study using the device with only the RA lead.

There are some unexplained mechanisms of AF termination by the reactive ATP. Delayed termination of AF may suggest some unidentified factors for the maintenance and termination of AF. Our study also indicated that immediate intervention of the A-ATP, which was programmed to “0 min” from the default “10 min” had a higher AF termination rate. However, we could not exclude the possibility that a higher A-ATP termination rate at “0 min” may be due to that including AF episodes with spontaneous termination within 10 min. Regarding battery consumption, if a patient continues to receive ATP therapy at a rate of 1000 sequences per month, it decreases the device longevity by only about 2.5%. The advantages of AF termination may outweigh the disadvantages of battery consumption. The optimal settings of the reactive ATP for the efficient suppression of AF will be determined in future studies.

Although the potent rhythm control strategy for AF is catheter ablation, not all patients are eligible for this invasive intervention. Reactive ATP is an option for suppressing AF especially in patients not eligible for catheter ablation. If A-ATP could terminate AF in a short time, it would be expected to reduce the risk of a stroke. Large prospective studies are required in the near future to disclose the details of the A-ATP.

### Study limitations

This was a single-center, retrospective observational study and the number of patients was small. We could not identify other confound clinical factors for a high ATP success rate. Furthermore, this study was not a randomized prospective study, which might have affected the results. We could not show a better prognosis for those patients as was shown in the MINERVA study due to the small sample size and short follow-up period. Further prospective, multicenter studies, including a larger number of patients with cardiac devices, are needed to confirm the detailed efficacy of the reactive ATP.

## Conclusions

Our study showed AF was frequently observed in patients implanted with CIEDs. The reactive ATP successfully terminated AF episodes in a certain number of patients. The termination rate was higher in the regular or slower atrial rate but lower in the irregular or faster atrial rate. Also, the immediate intervention of the A-ATP, which was programmed to “0 min” was superior to “10 min” for AF termination rate. Determining the optimal settings for this function may lead to further improvement in terminating AF episodes in these patients.

## Data Availability

The datasets generated and analyzed during the current study are available from the corresponding author on reasonable request.
